# A retrospective study on prophylactic regional lymphadenectomy versus nodal observation only in the management of dogs with stage I, completely resected, low-grade cutaneous mast cell tumors

**DOI:** 10.1186/s12917-021-03043-0

**Published:** 2021-10-15

**Authors:** Silvia Sabattini, Matti Kiupel, Riccardo Finotello, Damiano Stefanello, Eugenio Faroni, Walter Bertazzolo, Ugo Bonfanti, Antonella Rigillo, Sara Del Magno, Armando Foglia, Luca Aresu, Matteo Gambini, Mario Caniatti, Laura Marconato

**Affiliations:** 1 Department of Veterinary Medical Sciences, University of Bologna, Ozzano dell’Emilia, Bologna, Italy; 2 Department of Pathobiology and Diagnostic Investigation, College of Veterinary Medicine, Michigan State University Veterinary Diagnostic Laboratory, East Lansing, USA; 3 Department of Small Animal Clinical Science, Institute of Infection, Veterinary and Ecological Science, University of Liverpool, Neston, UK; 4 Department of Veterinary Medicine, University of Milan, Milan, Italy; 5 MYLAV Veterinary Laboratory, Rho, Milan, Italy; 6 Department of Veterinary Sciences, University of Torino, Grugliasco, Torino, Italy

**Keywords:** Canine, Lymphadenectomy, Mast cell tumor, Observation, Stage I

## Abstract

**Background:**

While lymphadenectomy of metastatic lymph nodes (LNs) has been associated with improved outcome, the clinical utility of prophylactic lymphadenectomy in dogs with stage I cutaneous mast cell tumors (cMCTs) remains a controversial topic. To assess the therapeutic role of lymphadenectomy of uninvolved regional LNs, the long-term outcome of cMCT-bearing dogs with cytologically negative and surgically unresected regional LNs (observation only, OO) was compared with that of dogs with surgically resected and histologically negative regional LNs (prophylactic regional lymphadenectomy, PRL).

**Results:**

A retrospective analysis of 64 dogs with a low-grade, completely resected stage I cMCT was performed: 35 (54.7%) dogs were subjected to OO and 29 (45.3%) underwent PRL. Dogs were monitored for a median of 813 and 763 days in the OO group and PRL group, respectively. The number of dogs undergoing MCT progression was significantly higher in the OO group (*P* = 0.028) and curve comparison revealed a tendency to a better time to progression in the PRL group (*P* = 0.058). No significant difference in survival time (*P* = 0.294) was observed between dogs in the OO and PRL groups.

**Conclusions:**

Our results showed that lack of immediate lymphadenectomy was associated with a higher risk for tumor progression. This preliminary judgement, reinforced by the findings that lymphadenectomy was well tolerated in all cases, and that histopathology provides the definitive assessment of the nodal pathological status, may suggest that prophylactic lymphadenectomy is indicated in the management of stage I MCTs. Larger prospective studies are warranted for generating clinical evidence of this latter hypothesis.

## Background

In canine cutaneous mast cell tumors (cMCTs), lymphatic drainage from the primary tumor has long been recognized as the most common initial route of metastatic spread, with the first site of metastasis identified as the draining nodal basin [1–3].

Prophylactic lymphadenectomy refers to the complete dissection of lymph nodes in human and veterinary patients with no evidence of nodal involvement [4–6]. While the therapeutic effect of regional lymphadenectomy has been documented in dogs with stage II cMCTs, the role of prophylactic regional lymphadenectomy in animals with stage I disease remains a controversial topic [7]. More often, dogs present with a clinically non aggressive cMCT and a cytologically negative lymph node (LN). It is difficult to advise owners about the need to surgically remove the LN alongside the primary tumor, as the amount and quality of information currently available does not offer a definitive answer to the question of the prognostic effect of prophylactic regional lymphadenectomy in early stage cMCTs.

Both an elective lymphadenectomy and a watchful-waiting policy have their proponents. The suspected high incidence rate of undetected or late LN metastasis in cMCTs is the main argument in favor of prophylactic lymphadenectomy, which is based on the rationale that further metastatic spread could be prevented at the level of the regional LN by eliminating the potential first neoplastic reservoir [8].

Conversely, the main arguments against prophylactic lymphadenectomy include the morbidities from the procedure including risk of lymphedema, increased length of surgery and complications from wound healing with unclear benefit, and the interference with the protective immune response to metastatic disease by removal of unaffected regional LNs [9, 10]. Such evidence derives from human medicine only, where patients undergo massive nodal dissection and immunity studies have been performed.

Additionally, the recent introduction of sentinel LN mapping in the diagnostic work-up of cMCTs has introduced the question regarding the clinical usefulness of prophylactic regional lymphadenectomy. According to three recent studies, the sentinel LN was different to the regional LN in 25–60% of dogs with cMCT [3, 11, 12].

Another problem of utmost importance concerning prophylactic lymphadenectomy indication is related to the diagnostic methods to classify a dog as node-positive or negative. The clinical examination, upon which the WHO classification is based, is far from being accurate, as palpation as well as imaging studies are unreliable predictors of nodal metastasis by themselves [13, 14].

It has been recently shown that non-palpable or normal-sized LNs may harbor metastatic disease and approximately 50% of those nodes either had early metastatic (HN2 according to the Weishaar classification) or overtly metastatic (HN3) disease, whereas the other half of dogs had nodes with no evidence of metastatic disease (HN0) or minimal suspicion of metastasis (HN1) [8, 15].

Due to the above, cytologic evaluation of the regional LN is always advised for the assessment of metastatic involvement [1]. Fine-needle aspiration (FNA) of the regional LN has been established as a cost-effective diagnostic tool to screen dogs for metastatic disease [16, 17].

Until 2009, the reporting and interpretation of LN cytology had caused considerable confusion in comparing results from different settings [18]. The introduction of the Krick criteria provided the opportunity to establish standard terminology and reporting guidelines for different diagnostic categories [16]. Based on cytology, five categories associated with escalating risk of malignancy have been proposed: “normal LNs”, “hyperplastic LNs”, “possible”, “probable” and “certain” metastasis based on the number of mast cells and the number and size of mast cell aggregates [16]. However, not unexpectedly, cytologic diagnosis of nodal metastasis may yield false-positive or false-negative results, leading to > 25% of discrepant cases when cytology and histology are compared [17, 19].

Another classification has been proposed by Weishaar et al. to standardize the histological assessment of metastatic involvement in dogs with cMCTs [15]. This system was found to correlate with clinical outcome in the original study [15]. However, labeling of the categories HN1 and HN2 is misleading, as they indicate stages of disease progression rather than degrees of a suspected diagnosis. Furthermore, the system is not based on a standardized trimming approach of examined nodes that may result in similar high false-positive and false-negative results as the cytologic system [15, 20, 21]. Regardless, the question that arises is whether LNs with no to rare (0–3), scattered, individualized mast cells in sinuses and/or parenchyma (HN0) or greater than three individualized mast cells in sinuses and/or parenchyma in a minimum of four high-power fields (HN1) represent no metastatic disease or whether dogs with such nodes will go on to develop macroscopic disease, stressing a different biology at play [22]. If HN0/HN1 LNs are essentially clinically and prognostically insignificant, lymphadenectomy could be targeted to only those LNs harboring metastatic disease (HN2/HN3). This would, however, require accurate determination of nodal metastasis without lymphadenectomy.

Thus, to investigate whether removal of potential clinically occult metastatic disease is associated with improved outcome, we first carried out an agreement study aimed at assessing the concordance between Krick’s cytological classification and Weishaar’s histological classification in diagnosing a LN as non-metastatic. Then, we retrospectively compared dogs with stage I, completely resected, low-grade cMCTs undergoing prophylactic regional lymphadenectomy (PRL group) with those where the regional LN was only monitored over time (observation only group, OO group).

We hypothesized that PRL provides a clinical benefit and is well tolerated.

Informed consent was obtained from animal owners for using data for the research purpose. Since this was a retrospective study, no approval from the Ethical Committee was required.

## Results

### Agreement study

Eighty-two cMCT-bearing dogs with cytologically negative regional LN undergoing subsequent lymphadenectomy and histological examination were reviewed: 48 (58.5%) LN aspirates were interpreted as normal and 34 (41.5%) as reactive according to Krick’s cytological evaluation. On the original histopathology reports, 48 (58.5%) LNs were interpreted as non-metastatic (HN0), 30 (36.6%) as pre-metastatic (HN1) and 4 (4.9%) as early metastatic (HN2). The negative predictive value of cytology in the identification of cMCT nodal metastases was 95.1%. This was considered sufficient to confirm the reliability of cytology in the identification of dogs without LN metastasis and to perform the subsequent clinical study.

### Clinical study

#### Patients and tumor characteristics

Overall, 64 dogs were included in the analysis. The most represented breeds were Labrador retriever (*n* = 17, 26.5%), Boxer (*n* = 10, 15.6%) and American Staffordshire terrier (*n* = 4, 11.4%). Of the remaining dogs, 10 were mixed-breed dogs, and 23 were breeds that were represented once or twice.

Median age was 7 years (range, 1–13) and median weight was 30.7 kg (range, 5–55). There were 36 females (29 spayed) and 28 males (15 neutered).

Tumors were located on limbs (*n* = 27; 42.2%), head and neck (*n* = 16; 25%), trunk (*n* = 13; 20.3%), inguinal region (*n* = 5; 7.8%) and mammary region (*n* = 3; 4.7%). Median maximum tumor diameter was 1.3 cm (range, 0.3–5.4); 60 (93.7%) cMCTs were not ulcerated, while 4 (6.3%) were.

All dogs were asymptomatic at presentation.

Based on the Patnaik grading system, there were 8 (14.3%) grade 1 cMCTs, and 56 (85.7%) grade 2 cMCTs. All were Kiupel low-grade.

Twenty-nine (45.3%) dogs had undergone PRL, whereas 35 (54.7%) had been subjected to OO.

In the PRL group, the following LNs were removed: inguinal (*n* = 10; 34.5%), superficial cervical (*n* = 7; 24.1%), submandibular (*n* = 6; 20.7%), popliteal (*n* = 5; 17.2%) and axillary (n = 1, 3.4%). Among them, 19 (65.5%) LNs were interpreted as non-metastatic (HN0) and 10 (34.5%) as pre-metastatic (HN1).

In the OO group, the following LNs were sampled: superficial cervical (*n* = 13; 37.1%), inguinal (*n* = 9; 25.7%), popliteal (*n* = 5, 14.3%), axillary (*n* = 4, 11.4%), and submandibular (n = 4, 11.4%). Among them, 27 (77.1%) LN aspirates were interpreted as normal and 8 (22.9%) as reactive.

The only difference among groups regarding demographic features and possible prognostic variables was a tendency towards a proportion of breeds predisposed to low-grade cMCTs in the OO group (Table 1).

**Table 1:** Demographic information, distribution of variables potentially associated with prognosis and follow-up information of 64 dogs with stage I low grade mast cell tumor treated by surgical excision and prophylactic regional lymphadenectomy or nodal observation only. Differences in data distribution were assessed with Chi-square test/Fisher’s exact test (categorical variables) or Mann-Whitney U test (quantitative variables)

Variable	Observation only (*n* = 35)	Prophylactic regional lymphadenectomy (*n* = 29)	***P***
Purebred			0.164
*Yes*	32	22	
*No*	3	7	
Breed			0.078
*Boxer, French bulldog, Weimaraner, Pug, American Staffordishire terrier*	13	5	
*Other*	22	24	
Age (years)			0.224
*Median (range)*	6.0 (2.0–11.0)	7.0 (1.0–13.0)	
Weight (kg)			0.237
*Median (range)*	33.0 (8.4–50.4)	27.4 (5.0–55.0)	
Sex			0.454
*Male*	17	11	
*Female*	18	18	
Neutering status			0.175
*Yes*	27	17	
*No*	8	12	
Anatomic location			0.137
*Head and neck*	8	8	
*Trunk and limbs*	25	15	
*Inguinal/perineal/mammary/digital*	2	6	
Tumor diameter (cm)			0.567
*Median (range)*	1.4 (0.5–5.4)	1.3 (0.3–9.0)	
Ulceration			> 0.999
*Yes*	2	2	
*No*	33	27	
Follow-up time (days) *Median (range)*	813 (290–2900)	763 (181–2039)	0.267
Disease progression			0.028*
*Yes*	6	0	
*No*	29	29	
Development of new MCT			0.037*
*Yes*	12	3	
*No*	23	26	
MCT related death			0.245
*Yes*	3	0	
*No*	32	29	

#### Treatment and outcome

In the OO group, the median follow-up time was 813 days (range, 290–2900). Overall, 6 dogs (17.1%) experienced cMCT progression after a median of 822 days (range, 560–1380): 4 (11.4%) experienced local relapse, 2 (5.7%) experienced nodal metastases in the LNs that had been previously aspirated, and 1 (2.9%) developed visceral metastasis. Median TTP was not reached. Twelve (34.3%) dogs developed new cMCTs after a median of 734 days (range, 197–1409) at the following locations: trunk (*n* = 6), limbs (*n* = 5), head and neck (*n* = 1).

At the end of the study, 25 (71.4%) dogs were alive, 7 (20%) had died because of cMCT-unrelated causes (*n* = 3 cardiac failure, *n* = 1 adrenal carcinoma, n = 1 acute lymphoblastic leukemia; n = 1 splenic hemangiosarcoma; n = 1 mesothelioma), and 3 (8.6%) had died because of cMCT-related causes after 1215, 1300 and 1471 days. Median ST was not reached.

In the PRL group, surgical complications related to lymphadenectomy did not occur, and no longer hospitalization was required compared with dogs undergoing surgical resection of the primary tumor only. The median follow-up time was 763 days (range, 181–2039). None experienced cMCT progression. Three dogs (10.3%) developed de novo cMCTs at the trunk after 321, 417 and 1092 days.

At the end of the follow-up period, 28 (96.6%) dogs were alive, and 1 (3.4%) had died because of tumor-unrelated causes after 835 days (metastatic anal sac carcinoma).

**Fig. 1:**
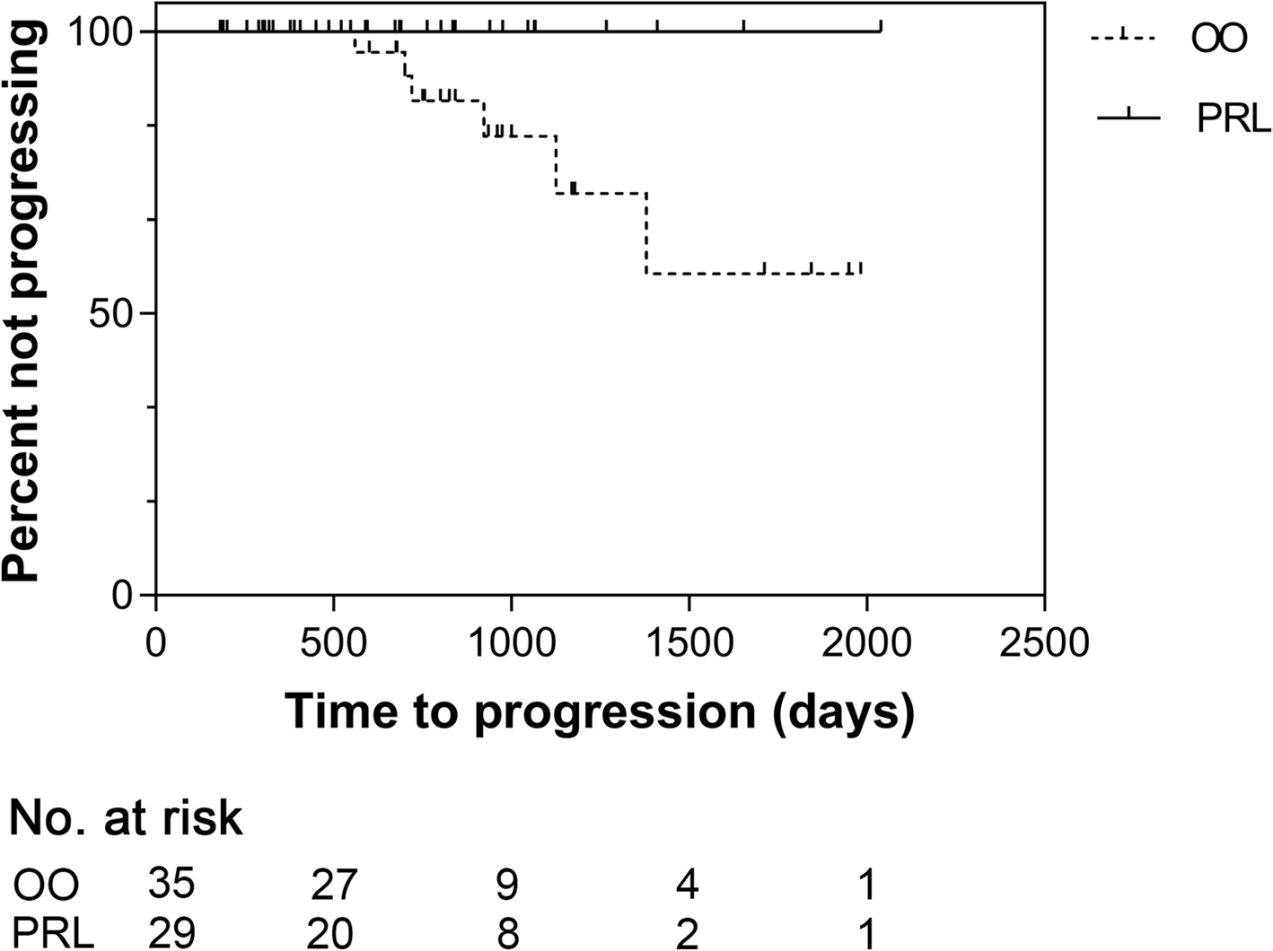
Time to progression for 64 dogs with surgically-removed stage I low grade mast cell tumor undergoing prophylactic regional lymphadenectomy (PRL, solid line) or regional lymph node observation only (OO, dashed line). Difference not statistically significant (*P* = 0.058)

The number of dogs undergoing cMCT progression was significantly higher in the OO group (*P* = 0.028), although no significant difference was observed in TTP (*P* = 0.058; Fig. 1; Table 1). Similarly, the number of dogs developing new cMCTs was significantly higher in the OO group (*P* = 0.037; Table 1).

**Fig. 2:**
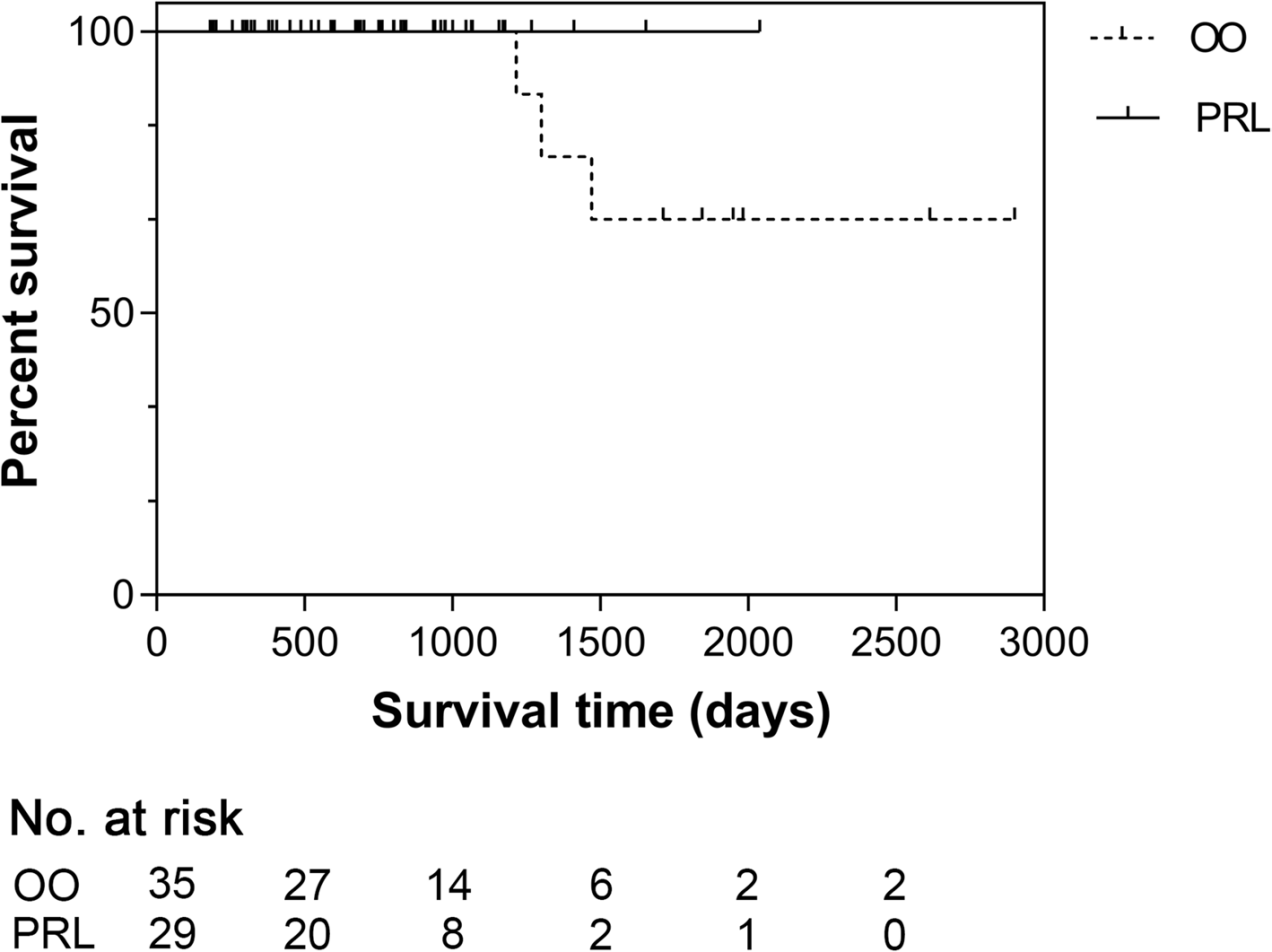
Survival time for 64 dogs with surgically-removed stage I low grade mast cell tumor undergoing prophylactic regional lymphadenectomy (PRL, solid line) or regional lymph node observation only (OO, dashed line). Difference not statistically significant (*P* = 0.294)

No significant difference in ST (*P* = 0.294) was observed between dogs in the OO and PRL groups (Fig. 2).

On Cox proportional hazards regression analysis, no factor was significantly associated with an increased risk of cMCT progression or cMCT-related death (Table 2).

**Table 2:** Univariable Cox regression analysis of variables potentially associated with increased risk of tumor progression and tumor-related death in 64 dogs with low-grade, completely resected stage I cMCT

Variable	Tumor progression		Tumor-related death	
	HR (95% CI)	*P*	HR (95% CI)	*P*
Breed predisposed to biologically aggressive cMCTs	0.92 (0.16–5.4)	0.925	0.39 (0.03–4.39)	0.443
Age > 7 years	4.02 (0.75–21.44)	0.104	6.3 (0.57–69.79)	0.133
Weight > 30.7 kg	1.38 (0.25–7.71)	0.714	0.99 (0.87–1.12)	0.832
Male sex	0.66 (0.12–3.62)	0.634	0.7 (0.06–7.81)	0.770
Neutering status	0.42 (0.08–2.07)	0.285	0.18 (0.02–2.01)	0.163
Biologically aggressive anatomic location	1.51 (0.18–13.01)	0.705	NA	
Tumor diameter > 1.3 cm	0.56 (0.10–3.09)	0.510	2.41 (0.22–26.59)	0.473
Ulceration	2.71 (0.29–25.30)	0.383	NA	
Patnaik grade 2	0.41 (0.05–3.59)	0.421	0.35 (0.03–3.88)	0.39
Lack of prophylactic regional lymphadenectomy	43.02 (0.04–41,697.1)	0.284	33.17 (0–1,727,740.46)	0.527

cMCT progression and cMCT-related death were not affected by Krick cytological LN score (normal or reactive) in the OO group (*P* = 0.46 and *P* = 0.838, respectively), or by Weishaar histological LN score (HN0 or HN1) in the PRL group (analysis not performed due to absence of events).

## Discussion

Over the past decade, new treatments for canine cMCTs have been developed [23]. Nevertheless, it is the authors’ opinion that the significant uncertainty in staging work-up and the considerable variability in current practice, mainly due to the lack of prospective evidence, have led to the unstandardized management of locoregional disease. While lymphadenectomy is the current standard approach for clinically suspected or positive LNs, regardless of histological grade of the primary tumor [7, 24], whether clinically unaffected LNs should undergo prophylactic regional lymphadenectomy when the primary cMCT is resected or whether only the primary cMCT should be resected remains a dilemma. Our results overall showed no significant differences in ST between operated dogs and those undergoing OO. However, a significantly higher proportion of dogs developing tumor progression and new cMCTs was observed in the group of dogs not receiving an elective regional LN dissection as part of their primary therapy.

As a general rule, an accurate preoperative diagnosis and strict follow-up are required to provide an adequate surgical dose while ensuring the therapeutic effect by narrowing down the target based on the risk–benefit balance. In other words, when it comes to surgical management, based on the current evidence, the extent of LN dissection should be adapted to clinical stage, as this corresponds to metastatic spread. To do so, several critical aspects need to be taken into consideration.

First, the identification of pathologically negative LNs contributes to the problem. Peripheral LNs are initially evaluated by means of physical examination and cytology, and a high degree of inaccuracy for these methods has been documented in the literature [8, 13, 19]. While the ultimate goal of FNA is to obtain cytologic material sufficiently to render a diagnosis of metastatic or non-metastatic LNs confidently, based on the current literature, the proportion of cytologically negative, histologically positive cases ranges from 10 to 50% [8, 17, 19].

In the current study, 82 cytologically negative nodes from the same institutions with the available corresponding histological reports on the surgical sample were retrospectively reviewed, yielding a false negative rate of approximately 5%. Also, 37% of the LNs were histologically pre-metastatic and not identified by cytology, underscoring the limits of this analysis.

Second, the indications for PRL remain subjects of much debate, since there are widely divergent views concerning the efficacy of routine lymphadenectomy and no evidence-based guidelines.

The argument in favor of PRL is based on the possibility that clinically or even histologically normal nodes may contain isolated malignant cells which, if not removed, may worsen outcome [7]. It is hypothesized that neoplastic cells may lie dormant in the regional LN for a considerable amount of time, only to recur or spread at a later point. This phenomenon has been well documented in human patients with melanoma, and PRL of the sentinel LN is recommended for selected patients [25, 26].

Conversely, removing LNs that appear unaffected may be unnecessary and potentially harmful, considering the higher morbidity associated with the procedure, increased duration of surgery and costs. Also, considering the host-tumor immunologic relationships, there may be concern raised for routinely removing unaffected LNs. Indeed, the extirpation of an immunologic defense organ may alter the host response to the tumor [10].

In the current series, lymphadenectomy was well tolerated, with no reported surgical complications. Additionally, even if this study failed to demonstrate a survival benefit for dogs undergoing lymphadenectomy compared with the nodal observation group, among the operated cases there was a reduced incidence of cMCT progression and new cMCT development. The first observation seems to be in line with the previously reported hypothesis that PRL might eliminate a potential neoplastic reservoir, representing at the same time a safe clinical procedure without evident complications. Indeed, in the current study HN1 LNs were considered as uninvolved, but still they contain an increased number of mast cells compared to normal nodes, which could represent a micrometastatic load rather than a reactive mast cell proliferation [15, 27].

It must be stressed that, according to our agreement study, 5% of dogs had a cytologically uninvolved LN, yet an early metastatic disease (HN2) based on histopathology. Also, the cytological slides were analyzed by board-certified clinical pathologists, which is not always performed in clinical practice, at least in our country. As a consequence, the false-negative results may be higher if the LN cytology slides are not sent to experienced clinical pathologists. Additionally, if the LNs are not palpable, they may not undergo cytological evaluation, so the nodal status is often unknown at the time of surgery. Because the therapeutic role of HN2 LN extirpation has been previously demonstrated [7], and due to the lack of complications related to lymphadenectomy [4, 8, 11], to recommend this additional surgical procedure may not be viewed as unnecessary or harmful. On the contrary, it may provide a clinical benefit, as shown by the reduced incidence of MCT progression found in the current series.

The finding that dogs undergoing lymphadenectomy of clinically uninvolved LN also had a reduced risk of de novo cMCT development is more difficult to explain. The most plausible explanation is that dogs in the OO group were more likely to develop new cMCTs, as predisposed breeds were more represented. More speculatively, it may be hypothesized that quiescent neoplastic cells residing in the regional LN may at some point regain the cell cycles and relocate at distant cutaneous sites, giving rise to overt disease [28]. In the current series, it was not investigated whether the new cMCTs were of the same histological grade and mutational status of the primary tumor, impeding any further comment.

In both groups the median ST was not reached, therefore it cannot be excluded that a survival advantage in either group may emerge with longer follow-ups. A power analysis was not performed but, due to the overall favorable prognosis for dogs with stage I low-grade cMCTs, very few tumor related events are to be expected, thus a very large number of cases would be needed to find a difference.

Several limitations of this study should be noted.

First, dogs did not undergo sentinel LN removal and, as a result, this study may have misdirected their lymphadenectomy in 25–60% of cases [11, 12]. As a consequence, it cannot be excluded that the extirpation of the sentinel rather than the regional LN would have improved outcome. Additionally, lymphocenters sometimes consist of more than a single LN. Therefore, it is possible that the entire lymphocenter was not removed during the lymphadenectomy, leaving additional regional LNs behind.

Second, the histological classification of HN0/HN1 nodes may have been impacted by the number of sections. Unlike human cancer pathology, there are currently no guidelines in veterinary medicine on how to section a LN and on how many sections need to be examined. In the current study, all LNs were sectioned along the major axis at the level of the hilus; thus, cell aggregates qualifying for HN2 nodal stage may have been missed.

Third, this study suffers the bias which are inherent to retrospective analysis. The surgeon’s decision as to whether to perform lymphadenectomy may depend not only on disease characteristics such as stage or histology, but also on the anatomic location, tumor size, or owner’s willingness. Consequently, dogs requiring a difficult surgical procedure (including, among others, axillary lymphadenectomy) would find themselves in a no-lymphadenectomy group, whereas those with an easily accessible cMCT and/or regional LN may undergo lymphadenectomy more commonly. Also, even if surgical complications related to lymphadenectomy were not reported, it may be possible that minor sequalae were not documented; also, no quality-of life assessment was carried out, potentially hiding disadvantages of the additional treatment burden.

Additionally, only dogs with completely resected, low-grade cMCTs were included in the study, and this information is often retrieved only after surgery. Nevertheless, provided that cytologic grading may help predicting the histological grade, due to the high rate of locoregional relapse, high-grade cMCTs will require lymphadenectomy in any case [4, 24, 29, 30].

For the agreement study, samples were not evaluated by all anatomic pathologists involved in the study, possibly biasing the results.

Last, even though the median follow-up time was not significantly different among groups, dogs undergoing OO were monitored for a median of 813 days versus a median of 763 days for dogs undergoing nodal dissection. It cannot be excluded that, with a longer follow-up in operated dogs, the outcome differences may cancel out.

In conclusion, whether regional dissection of clinically negative LNs should be part of the primary resection for cMCTs remains a problem of legitimate concern.

Our results showed that lack of immediate lymphadenectomy was associated with a higher risk for tumor progression. This preliminary judgement, reinforced by the findings that lymphadenectomy was well tolerated in all cases, and that histopathology provides the definitive assessment of the nodal pathological status, may suggest that prophylactic lymphadenectomy is indicated in the management of stage I cMCTs. Larger prospective studies are warranted for generating clinical evidence of this latter hypothesis.

## Material and methods

This retrospective multi-institutional study consisted of an agreement part and a clinical part.

The agreement study was aimed at assessing the consistency of cytology in the correct identification of uninvolved LNs, by testing the concordance between Krick’s cytological classification and Weishaar’s histological classification in diagnosing a LN as non-metastatic.

The clinical study was aimed at assessing the therapeutic role of PRL of grossly and cytologically unremarkable regional LNs in canine low-grade, completely resected cMCT. To do so, the long-term outcomes of MCT-bearing dogs with cytologically unremarkable and surgically unresected regional LN were compared with those of dogs with a surgically resected and histologically normal or minimal risk regional LN.

The regional LN was defined as the closest LN in the expected lymphatic drainage, and was identified either by palpation or by ultrasound [31].

Regional LNs were considered cytologically free of metastasis if classified as normal or with reactive lymphoid hyperplasia according to the scheme proposed by Krick [16]. They were considered histologically free of metastasis if classified as HN0 or HN1 according to the scheme proposed by Weishaar [15].

### Agreement study

For the agreement study, dogs with low grade cMCTs and cytologically negative (normal/reactive according to Krick’s criteria) [16] regional LNs undergoing subsequent lymphadenectomy and histological examination were identified from the same oncology centers participating in the clinical study. Two of the four centers had the cytologic and histologic preparations read out by internal board-certified veterinary anatomic pathologists. The remaining two centers had both cytological and histologic samples submitted to the same private laboratory and were read out by two board-certified clinical pathologists and two anatomic pathologists, respectively. All cytological preparations had been obtained by FNA, with or without ultrasound guidance using 27G or 25G needles. Smears were generated from obtained sample material and air-dried, and then stained with May Grünwald-Giemsa. All histological samples were fixed in 10% neutral-buffered formalin and paraffin-embedded following routine processing. Serial sections were cut and routinely stained with hematoxylin and eosin for histologic evaluation. Replicate sections were stained with toluidine blue or Giemsa to highlight mast cell granules. For each dog, the histological findings were correlated with the cytological findings, in order to evaluate the negative predictive value of cytology in the identification of MCT nodal metastases; i.e., the probability that dogs with a cytological result of an unremarkable LN will have a histologically unremarkable LN as well.

### Clinical study

For this part, the medical records of 4 oncology centers were reviewed from January 1st,2010 to January 1st, 2020 to identify dogs with treatment-naive, firstly occurring, completely resected, low-grade cMCTs. Such dogs were further divided into dogs with [1] non surgically resected regional LNs (OO group) that were cytologically-negative (normal/reactive according to Krick’s criteria) [16] or dogs with [2] surgically resected regional LNs (PRL group) that were histologically-negative (HN0/HN1 according to Weishaar’s criteria) [15]. Decisions regarding whether to perform OO or PRL were made according to each clinician’s discretion.

Tumors were defined as completely resected in the absence of neoplastic cells within 2 mm from surgical margins, assessed microscopically on sections obtained by the breadloaf-cross method.

To be eligible for recruitment, dogs had to undergo complete staging and surgical excision of the primary cMCT, consisting of wide lateral surgical margins of 3 cm and one fascial plane deep, and have a minimum follow-up of 180 days. Information on clinical stage was obtained by means of hematological and biochemical analysis, cytological evaluation of the cMCT and regional LN, thoracic radiographs, abdominal ultrasound, and FNA of liver and spleen.

Dogs with recurrent, concurrent multiple, subcutaneous or high-grade MCTs or those with stage II-IV disease were excluded from the study. Also, dogs were excluded if they had received neoadjuvant or adjuvant antitumoral treatment, and if the cMCTs had been removed with incomplete margins.

Background information recorded for each dog included: signalment; cMCT description (location, size, presence of ulceration); clinical substage; date of surgery; local relapse (defined as the cytological evidence of a recurrent cMCT within 2 cm from previous scar); nodal relapse (defined as the development of cytologically or histologically-confirmed LN metastases); distant relapse (defined as the occurrence of visceral metastasis); development of de novo cMCTs (defined as the occurrence of a new cMCT at a distant cutaneous site having a different regional LN), date of death or last follow-up examination, and cause of death.

The characteristics of relapse (local, nodal and distant) and the survival impact were compared between the OO and PRL groups.

Regardless of the group, dogs were monitored post-operatively by means of clinical examination, blood testing, and abdominal ultrasound, performed every 3 months during the first year, and every 6 months thereafter. In case of progression of any type, dogs underwent a complete re-staging.

### Statistical analysis

Descriptive statistics were used in the analysis of dogs and tumor characteristics. When appropriate, data sets were tested for normality with the Shapiro-Wilk test. Values were expressed as mean ± SD in case of normal distribution, or as median with a range in case of non-normal distribution.

The distribution of demographic features and possible prognostic variables between the OO and PRL groups were assessed with Student’s T-test (quantitative, parametric variables), the Mann-Whitney *U* test (quantitative, non-parametric variables) or the Chi-square test/Fisher’s exact test (categorical variables). Chi-square was calculated if all expected cell frequencies were equal to or greater than 5.

The considered variables included breed (purebred and predisposition to biologically aggressive cMCTs, that is, Shar-pei, Labrador retriever and Golden retriever; and predisposition to low-grade cMCTs, that is, Boxer, French Bouledogue, Weimaraner, Pug, American Staffordshire terrier), age, body weight, sex, neutering status, anatomic site of the primary cMCT (head and neck, trunk and limbs, inguinal/perineal/mammary region and digits), substage, macroscopic tumor largest diameter (measured with a digital caliper), ulceration, and development of new cMCTs.

The influence of the above variables and of lymphadenectomy on cMCT progression and cMCT- related death was investigated with Cox proportional hazards regression analysis. The impact of Krick cytological LN score (normal or reactive – OO group) and Weishaar histological LN score (HN0 or HN1 – PRL group) on cMCT progression and cMCT-related death were also assessed with the same methods. Survival plots were generated according to the Kaplan-Meier product-limit method. For survival analyses, quantitative variables (age, weight and tumor diameter) were dichotomized using the median value as cut-off.

Time to cMCT progression (TTP) was calculated from the date of surgery to the first occurrence of one or more of local, nodal or distant relapse. Dogs with no recurrence or disease progression at the date of the last visit or death were censored.

cMCT-specific survival time (ST) was calculated from the date of surgery to the date of death or to the date of the last visit if death did not occur. Only dogs deceased for cMCT-related causes were considered as events.

Data were analyzed by use of commercial software programs (SPSS Statistics v. 19, IBM, Somers, New York, and Prism v. 5.0, GraphPad, San Diego, California). *P* values ≤.05 were considered significant.

## Data Availability

The datasets generated and/or analyzed during the current study are available from the corresponding author on reasonable request.
